# Colorectal leiomyosarcoma: A case report^☆^

**DOI:** 10.1016/j.radcr.2022.05.023

**Published:** 2022-06-07

**Authors:** Elias Lugo-Fagundo, Elliot K. Fishman

**Affiliations:** Johns Hopkins Medicine, The Russell H. Morgan Department of Radiology and Radiological Science, 601 North Caroline Street, Baltimore, MD 21287, USA

**Keywords:** Leiomyosarcoma, Gastrointestinal stromal tumors, Smooth muscle tumor, GI tract

## Abstract

Leiomyosarcomas are rare aggressive smooth muscle tumors that represent 0.1% of all colorectal malignancies. The lack of literature available concerning leiomyosarcomas presents a challenge when diagnosing and treating these tumors, thus it is crucial that we differentiate them from gastrointestinal stromal tumors (GIST), the most common type of mesenchymal neoplasms of the gastrointestinal (GI) tract, especially when considering leiomyosarcoma's high prevalence of recurrence and malignancy. In this article, we present a case of a 74-year-old male with a diagnosis of colonic leiomyosarcoma. We analyze the tumor's CT imaging findings as well as correlation with the patient's pathological findings including immunostains, size, and mitotic activity, as well as the patient's risk for recurrence.

## Introduction

Gastrointestinal stromal tumors (GIST) are the most prevalent type of mesenchymal neoplasms in the gastrointestinal (GI) tract [1]. However, it wasn't until the late 1990s when Hirota et al. introduced the presence of KIT-mutations, which allowed for a distinction between GIST and leiomyosarcomas to be made [2]. Since its differentiation in 1998, leiomyosarcoma, a true smooth muscle tumor, has accounted for less than 0.1% of all colorectal malignancies, which may account for the dearth of literature discussing the tumor's demographic, radiologic, and clinicopathological features. Proper diagnosis of leiomyosarcoma is critical given the tumor's aggressiveness and potential for recurrence [3,4]. In this article, we report the case of a 74-year-old male with a diagnosis of colonic leiomyosarcoma and correlate the imaging and pathological findings.

## Case report

**Fig. 1:**
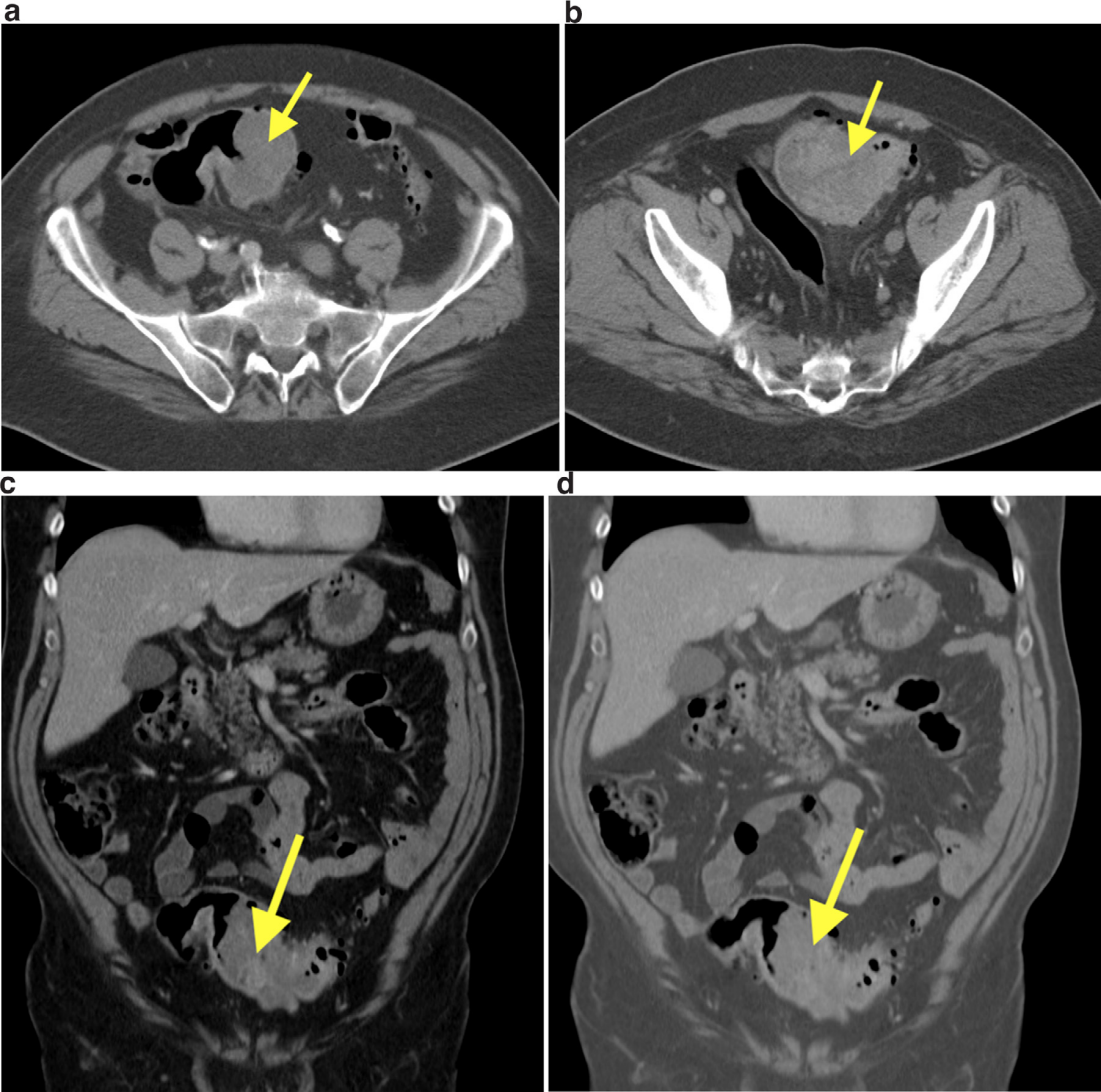
(A,B) CT scan done with IV contrast material demonstrates a 10 cm mass (arrows) in the sigmoid colon with areas of tumor necrosis but no perforation. (C,D) CT scan in coronal plane and 3D volume rendering demonstrates the lobular mass (arrows) that has areas of necrosis. The tumor is not obstructing the bowel.

A 74-year-old male presented to his local hospital with complaints of persistent diarrhea over the previous 2 months, as well as intermittent rectal bleeding. An obstructing mass that could not be traversed was identified via colonoscopy. A follow-up computed tomography (CT) scan displayed a mass-like dilatation involving the short segment of the sigmoid colon, composed of heterogeneous soft tissue density, measuring 10 × 6 × 9 cm Fig. 1. The mass distended the sigmoid colon (IA) but there was no evidence of bowel obstruction. The coronal and 3D volume-rendered views define that the mass distended but did not obstruct the colon. No perforation on hemorrhage was seen. As a result, the patient underwent an urgent low anterior resection of the rectosigmoid colon at an outside institution. Following the procedure, pathology revealed an 8.7 cm grade 3 leiomyosarcoma involving all layers of the bowel wall. Submitted immunostains showed that neoplastic cells were negative for LCA, MART1, pankeratin, CD117, DOG1, and S100, and reactive for desmin and SMA (weak). Twelve lymph nodes were negative for tumor; however, 48 mitoses were identified in 5 mm^2^, and less than 50% tumor necrosis was observed.

A follow-up positron emission tomography (PET) scan indicated some residual tumor at the site of the colectomy, as well as a borderline enlarged left upper paratracheal lymph node. Given a review of the available literature, gemcitabine/taxotere chemotherapy was selected as the main course of treatment. Additionally, due to the patient's concerning PET scan images and the tumor's high rate of recurrence, we advised close follow-up and regular imaging.

## Discussion

This article reviews a case of colonic leiomyosarcoma, an extremely rare smooth muscle tumor commonly found in the stomach, followed by the small intestines, colon, and rectum [5]. Smooth muscle tumors in the GI can be further classified into 2 distinct subgroups: tumors smaller than 10 cm with mitotic rates of less than 3 per 5 mm^2^ are labeled as leiomyomas, and tumors larger than 10 cm with mitotic rates of 3 or greater per 5 mm^2^ as leiomyosarcomas [1]. Prior to the identification of the oncogenic role of KIT, a tyrosine kinase receptor proto-oncogene, in GIST, the majority of GI mesenchymal malignancies were misdiagnosed as leiomyosarcomas, leiomyoblastomas, or leiomyomas [6]. However, given the poor prognosis and lack of literature associated with leiomyosarcomas, as well as the clinical and histological resemblance between both tumors, it is critical to properly differentiate between both malignancies. Diagnosis of GIST, which originate in the interstitial cells of Cajal, relies on the immunohistochemical positivity for CD117, CD34, and DOG1. Conversely, smooth muscle tumors demonstrate immunohistochemical negativity for CD117, CD34, DOG1, and S100, as well as positivity for SMA and desmin, all of which are consistent with the immunostains from the case we have presented; additionally, they lack KIT and PDFFRA mutations [3,5,7].

We report a 74-year-old male who presented to his local hospital with frequent diarrhea and occasional rectal bleeding. Leiomyosarcomas present various non-specific symptoms including rectal and intra-abdominal bleeding, abdominal pain, weight loss, diarrhea, tenesmus, constipation, bowel obstruction, and fever [8]. Following resection of the rectosigmoid colon, the treatment of choice for GI leiomyosarcomas and other soft tissue tumors [9], and immunostaining of the sigmoid mass, the tumor was identified as a leiomyosarcoma. Given the tumor's size (8.7 cm) and mitotic activity (48 per 5 mm^2^), the patient's risk of progressive disease was 86%; additionally, the high rate of tumor necrosis was consistent with aggressive leiomyosarcomas [1]. Unfortunately, a follow-up PET scan demonstrated residual tumor, and the patient was recommended for chemotherapy.

CT appearance of leiomyosarcomas in this case was noteworthy because despite its nonspecific features, it was helpful in outlining the mass and determining the viability of tumor resection.

## Patient consent

The patient reported in the manuscript signed the informed consent/authorization for participation in research, which includes the permission to use data collected in future research projects such as presented case details and images used in this manuscript.
